# Inhibiting Production of New Brain Cells during Puberty or Adulthood Blunts the Hormonally Induced Surge of Luteinizing Hormone in Female Rats

**DOI:** 10.1523/ENEURO.0133-17.2017

**Published:** 2017-11-02

**Authors:** Margaret A. Mohr, Lydia L. DonCarlos, Cheryl L. Sisk

**Affiliations:** 1 Neuroscience Program, Michigan State University, East Lansing, MI 48824,; 2 Department of Cell and Molecular Physiology, Stritch School of Medicine, Loyola University Chicago, Maywood, IL 60153

**Keywords:** anteroventral periventricular nucleus, LH surge, postnatal gliogenesis, postnatal neurogenesis, puberty

## Abstract

New cells are added during both puberty and adulthood to hypothalamic regions that govern reproduction, homeostasis, and social behaviors, yet the functions of these late-born cells remain elusive. Here, we pharmacologically inhibited cell proliferation in ventricular zones during puberty or in adulthood and determined subsequent effects on the hormone-induced surge of luteinizing hormone (LH) in female rats. Initial neuroanatomical analyses focused on verifying incorporation, activation, and pharmacological inhibition of pubertally or adult born cells in the anteroventral periventricular nucleus (AVPV) of the hypothalamus because of the essential role of the AVPV in triggering the preovulatory LH surge in females. We first showed that approximately half of the pubertally born AVPV cells are activated by estradiol plus progesterone (P) treatment, as demonstrated by Fos expression, and that approximately 10% of pubertally born AVPV cells express estrogen receptor alpha (ERα). Next, we found that mitotic inhibition through intracerebroventricular (ICV) administration of cytosine β-D-arabinofuranoside (AraC), whether during puberty or in adulthood, decreased the number of new cells added to the AVPV and the suprachiasmatic nucleus (SCN), and also blunted and delayed the hormone-induced LH surge. These studies do not prove, but are highly suggestive, that ongoing postnatal addition of new cells in periventricular brain regions, including the AVPV and SCN, may be important to the integrity of female reproduction.

## Significance Statement

We investigated the role of pubertally and adult-born hypothalamic cells in the generation of the ovarian hormone-induced luteinizing hormone (LH) surge, which triggers ovulation in female rodents. In the anteroventral periventricular nucleus (AVPV), the neural site where ovarian hormones induce the LH surge, many postnatally added cells become mature neurons or glial cells, many are activated by hormones during the surge, and some express estrogen receptor α (ERα). Importantly, pharmacological knockdown of cell proliferation in ventricular niches, either during puberty or in adulthood, disrupts the LH surge. This study suggests a novel mechanism for both the pubertal establishment and adult maintenance of female reproductive function, namely the incorporation of postnatally born neurons and glia into neural circuits essential for female fertility.

## Introduction

It is now indisputable that neurons and glia are added during both puberty and adulthood to limbic and hypothalamic regions that govern reproduction, homeostasis, and social behaviors, offering an unexpected mechanism for neural plasticity throughout the life span (Bernier et al., 2002; Rankin et al., 2003; Kokoeva et al., 2005; He and Crews, 2007; Mak et al., 2007; Ahmed et al., 2008; Antzoulatos et al., 2008; Fowler et al., 2008; Migaud et al., 2010; Cheng et al., 2011; Mohr and Sisk, 2013; Bless et al., 2014; Saul et al., 2014; Staffend et al., 2014; Migaud et al., 2015; Bless et al., 2016; Mohr et al., 2016; Jhaveri et al., 2017). Although clues about the function of these late-born cells can be gleaned from investigations of their molecular identities and the internal and external signals that activate them (Pencea et al., 2001; Matsuzaki et al., 2009; Pierce and Xu, 2010; Lee et al., 2012; Bless et al., 2014; Sousa-Ferreira et al., 2014; Bless et al., 2016; Chaker et al., 2016), it has been a challenge to directly link late-born cells with specific functions or behavior, in part because the relevant brain regions are associated with multiple functions.

Previous investigations of sex differences in late-born cell addition identified the anteroventral periventricular nucleus (AVPV) as a hypothalamic cell group in which there are more pubertally born cells in females than in males (Ahmed et al., 2008; Mohr et al., 2016). The AVPV performs a pivotal function in females: it is a nodal point for the integration of signals from the brain and ovaries that govern ovulation (Wiegand and Terasawa, 1982; Petersen and Barraclough, 1989). Estrogens and progestins, produced by the ovaries and the brain (Micevych and Sinchak, 2008; Terasawa and Kenealy, 2012), drive AVPV neurons to compel another small, scattered, and essential group of neurons to release a surge of gonadotropin-releasing hormone (GnRH) into the pituitary portal system, eliciting a surge of luteinizing hormone (LH), which then triggers ovulation. In rats, the GnRH surge is strictly timed by neural inputs to the AVPV from the suprachiasmatic nucleus (SCN), the primary central circadian clock (Williams and Kriegsfeld, 2012). Prepubertal female rats, adult females without a functioning AVPV, and male rats all have substantial GnRH, but are incapable of generating a hormone-driven GnRH surge from the brain, and therefore are incapable of mounting an LH surge from the pituitary (Wiegand and Terasawa, 1982; Petersen and Barraclough, 1989). The crucial function and relative tractability of the AVPV prompted us to ask whether pubertally born AVPV cells are functionally incorporated into reproductively relevant circuits, and more generally, is cell addition during puberty, or even during adulthood, critical to regulation of the adult GnRH/LH surge?

These questions address fundamental processes of brain development and maintenance of reproductive competence. Pubertally born cells may support the pubertal gain of function in neuroendocrine positive feedback in females, without which generation of the GnRH/LH surge may be impossible. Alternatively, cells born during puberty or adulthood may help maintain cyclic function throughout female reproductive life. An important clinical implication is that, if addition of new brain cells is important to female reproduction, whether during puberty or adulthood, then agents that alter this process in the brain, as well as the periphery, such as radiation or chemotherapeutics, may result in infertility via actions on the nervous system (Müller, 2002).

Here, we report a series of experiments that demonstrate hormone-driven activation of pubertally born AVPV cells, and, in a subset of these pubertally born cells, expression of estrogen receptor α (ERα), a critical link in generation of the GnRH and LH surge. Moreover, addition of new cells, not only to the AVPV, but also to the SCN, continues into adulthood in females. Reducing cell proliferation in ventricular zones, either during puberty or in adulthood, via intracerebroventricular (ICV) delivery of a mitotic inhibitor blunted and delayed the ovarian hormone-induced LH surge, suggesting a causal role for newly generated cells in adult female reproductive function.

## Materials and Methods

### Animals

Animals were treated in accordance with the National Institutes of Health Guide for the Care and Use of Laboratory Animals and with the approval of the Michigan State University Institutional Animal Care and Use Committee. Weanling [postnatal day 21 (P21)] and young adult (∼P60) Sprague Dawley female rats were ordered from Harlan and singly housed in 37.5 × 33 × 17 cm clear polycarbonate cages with ad libitum access to food (Teklad Rodent diet no. 8640; Harlan) and water. Animals were maintained on a 14/10 h light/dark cycle (lights off at 4 P.M.). Animals were given a week to acclimate after arrival before experimental manipulation, with daily handling.

### Experimental design and statistical analyses

General methodological details for animals, treatments, procedures, and microscopic analyses for the following experiments follow this section. All statistics were calculated using IBM SPSS Statistics, version 22.

#### Experiment 1. Are pubertally born AVPV cells activated (express Fos) during the hormonally induced LH surge?

Female rats received ICV infusions of the cell birth date marker 5-bromo-2’-deoxyuridine (BrdU) for four weeks, from P28 to P56. On P56, rats were ovariectomized (isoflurane anesthesia, bilateral flank incisions) and allowed to recuperate for one week. Seven rats were treated with estradiol and progesterone (P) to induce an LH surge, and six rats were treated with oil vehicle. On P65, ∼6 h after the P (or oil) injection, rats were perfused. Brain tissue from these animals was used to determine whether BrdU-immunoreactive (BrdU-ir, i.e., pubertally born) AVPV cells expressed Fos during the LH surge via double-label immunofluorescence. Group differences in the percentage of BrdU-ir cells that were also Fos-ir were assessed using a one-way ANOVA with a between subjects design, with the percentage of BrdU-ir cells expressing Fos in four anatomically matched sections of the AVPV as the dependent variable and treatment (oil vs hormones) as the independent variable. Cohen’s *d*, a measure of effect size, was calculated using the mean and SD for each group; effect size is considered large when Cohen’s *d* ≥ 0.8.

#### Experiment 2. Do pubertally born AVPV cells express ERα or P receptor (PR)?

ERα- and PR-expressing AVPV cells are critical for the ovarian hormone-induced LH surge (Radovick et al., 2012). Estradiol downregulates ERα in the preoptic area (DonCarlos et al., 1991; Simerly et al., 1996), but upregulates PR (DonCarlos et al., 1989; Simerly et al., 1996). Therefore, to optimize steroid receptor localization, one series of tissue sections from four ovariectomized, oil-treated rats in experiment 1 was used for double-label immunofluorescence for BrdU and ERα, and one series from four ovariectomized, estradiol and P-treated rats in experiment 1 was used for double-label immunofluorescence for BrdU and PR. The percentage of BrdU-ir cells that were either ERα- or PR-positive in four anatomically matched sections through the AVPV was calculated.

#### Experiment 3. Does pharmacological inhibition of cell proliferation during puberty or adulthood affect the hormone-induced LH surge?

Prior research showed that new cells are added to AVPV during the juvenile period and during early and mid-puberty (Ahmed et al., 2008), but whether postnatal addition of new cells to the rat AVPV extends beyond mid-puberty had not been investigated. To address this question, female rats received a four-week ICV infusion of the mitotic inhibitor cytosine β-D-arabinofuranoside (AraC) or vehicle control (which contained BrdU), either during puberty (four to eight weeks of age), or in young adulthood (9/10-13/14 weeks of age). Rats treated during puberty were monitored daily on arrival in the laboratory to determine the day of vaginal opening; all rats in this study were weighed daily throughout the experiment. After four weeks of ICV AraC or vehicle, rats were anesthetized with isoflurane, and minipumps were removed. Following removal of minipumps, vaginal smears were collected daily for two weeks from five rats in each of the four groups before ovariectomy at 10 (pubertal treatment) or 15–16 (adult treatment) weeks of age. The remaining rats in each group were ovariectomized at the time of removal of the minipumps (8 and 13 weeks of age, pubertal and adult treatments, respectively). At the time of ovariectomy, all rats were fitted with rat femoral vein tapered catheters (Alzet catalog number 0007745) for repeated blood sampling. After a 2–3 d recovery period, animals with patent catheters received hormone priming to induce an LH surge. After the P injection at 10 A.M., 200 μl blood samples were obtained hourly from 11 A.M. until 1 P.M., every half-hour from 1:50 to 3 P.M., and hourly from 3 to 7 P.M. At each blood collection, sterile saline (200 μl) was replaced via the catheter. Four to five days after the first induction of the LH surge, rats received hormone treatment to induce another LH surge, and were perfused 6 h after the P injection. Cannula placement was confirmed during brain sectioning; no animals had to be excluded from analyses for misplaced cannulas. For a time line of this experiment, see Figure 6*A*.

Brain sections through the AVPV were used for either single-label BrdU immunohistochemistry, double-label immunofluorescence to determine whether BrdU-ir cells expressed Fos, or triple-label immunofluorescence to determine whether BrdU-ir cells expressed markers of mature neurons (NeuN) or astrocytes [glial fibrillary acidic protein (GFAP)]. Animals with catheters that had lost patency could not be included in the analysis of the LH surge, but these animals were included in the tissue analyses for immunohistochemistry or immunofluorescence. The small number of AVPV sections available precluded performing double- and triple-label immunofluorescence in all rats, therefore these procedures were performed in a subset of rats from each group. Final sample sizes for the different analyses are reported below.

To determine whether AraC treatment affected LH concentrations across time in pubertal- and adult-treated animals, a two-way repeated measures ANOVA was performed with LH concentration as the within-subjects factor and treatment (AraC vs vehicle control) and age during treatment (puberty vs adulthood) as the between subjects factors (*n* = 4 pubertal-control; *n* = 3 pubertal-AraC; *n* = 4 adult-control; *n* = 4 adult-AraC). Significant effects were followed up by Fisher’s LSD. Area under curve calculations were performed to determine whether the size of the LH surge differed between animals treated with AraC during puberty or adulthood compared with age-matched controls. To determine whether AraC treatment affected the peak of the LH surge or the timing of the LH surge, the peak value of plasma LH was recorded along with the time that the peak occurred for each animal. Two-way ANOVAs (AraC/control and pubertal/adult treatment) were performed to determine whether AraC treatment affected the maximum LH concentration or the time of the LH peak. These ANOVAs revealed a main effect of treatment in both instances, and Cohen’s *d* was calculated for these main effects to estimate effect size.

To compare the number of AVPV cells added during puberty versus adulthood, and to assess the effectiveness of AraC in interrupting cell proliferation, a two-way ANOVA with a between subjects design was performed with age during treatment (puberty or adulthood) and treatment (AraC or vehicle control) as the independent variables and the total number of BrdU-ir cells in four anatomically matched 30μm sections of the AVPV as the dependent variable (*n* = 7 pubertal control; *n* = 5 pubertal AraC; *n* = 6 adult control; *n* = 7 adult AraC). This ANOVA revealed main effects of both age during treatment and treatment, and Cohen’s *d* was calculated for each of these main effects to estimate effect sizes.

Our finding that AraC treatment resulted in a ∼50 min delay of the LH surge (see Results) raised the possibility of SCN involvement, although neither we nor others that we know of had previously investigated whether cells are added postnatally to the SCN. Fortuitously, the series of sections used for single-label BrdU immunohistochemistry in this experiment included the two most rostral sections of the SCN, in most animals, and we quantified BrdU-ir cells in these sections as well (*n* = 5, all groups).

To examine whether the proportion of AVPV BrdU-ir cells that were activated during the surge differed in pubertal and adult vehicle control groups, a between subjects one-way ANOVA was performed with age during treatment (puberty vs adulthood) as the independent variable, and the percentage of BrdU-ir cells that were also Fos-ir in four anatomically matched sections of the AVPV as the dependent variable (*n* = 3/age). Qualitative assessment of BrdU/NeuN or BrdU/GFAP colocalization was explored in four anatomically matched sections of the AVPV in AraC- or vehicle-treated animals (*n* = 2/age/treatment). The percentage of BrdU-ir cells that were also NeuN-ir or GFAP-ir was calculated for each group but statistical analyses were not performed because of the small sample sizes.

### LH surge induction and timing

We conducted a pilot study to determine the timing of a hormonally induced LH surge in female rats under our laboratory conditions. Adult female Sprague Dawley rats (P60-P70; Harlan) were ovariectomized and fitted with femoral catheters with tethers, then 1 week later an LH surge was induced. To induce an LH surge, rats received injections of estradiol benzoate (EB; s.c., 10 μg in 0.05 ml sesame oil) or vehicle for two consecutive days at 10 A.M. On the third day, animals received P (s.c., 500 μg in 0.1 ml sesame oil) or oil at 10 A.M. Blood was collected (200 μl) every hour starting the morning of P treatment, at 8 A.M., then every 20 min from 11 A.M. to 1 P.M., then hourly until 3 A.M., for a total of 24 samples per animal. Plasma LH concentrations were determined via radioimmunoassay (RIA). After determining that the peak LH concentration was 35.1 ± 8.7 ng/ml at 3 P.M., ∼5 h following the P treatment, another LH surge was induced by EB and P treatment in four of the six rats used to determine the timing of the LH surge, and the remaining two received oil vehicle injections. All rats were perfused 6 h after P or oil treatment, approximately 1 h after the expected LH peak and when surge-induced Fos expression in the AVPV was expected to be maximal (Lee et al., 1990). Single-label Fos immunohistochemistry was performed and analyzed on sections through the AVPV as previously described (Romeo et al., 1998), but without nickel chloride in the diaminobenzidine solution. The density of Fos-ir AVPV cells was three to four times higher in rats given EB/P compared with rats given oil, confirming that EB/P induction of an LH surge is associated with increased Fos expression in the AVPV. We used the same regimen of hormone treatment in the current experiments to induce an LH surge; blood sampling and killing time points selected for the current experiments were based on the results of this pilot experiment.

### RIA for LH

Plasma LH concentrations were determined from duplicate 50 µl samples using Rat LH RIA kit from A. F. Parlow (Scientific Director, National Hormone and Peptide Program, Harbor-UCLA Medical Center) according to previously published methods (Sisk and Desjardins, 1986). The lower limit of detectability for the assay was 0.8 ng/ml; all values that fell below the assay sensitivity were adjusted to 0.8 ng/ml. The intraassay coefficient of variation was 11.0%. Samples from this assay that were above 30 ng/ml were re-run with either 10 or 25 µl plasma samples, with standard curves ranging from 1.6 to 60 or 4 to 150 ng/ml, respectively, and with an intraassay coefficient of variation of 6.6%.

### Surgeries and drug treatments

In experiments 1 and 2, rats underwent stereotaxic surgery to receive unilateral ICV cannulas (Alzet Brain Infusion Kit 2) aimed at the lateral ventricle (1.00 mm posterior, 1.2 mm lateral, and 4.00 mm ventral from bregma) and attached to osmotic minipump implants designed to deliver BrdU (Sigma; 4.5 µg/µl) in sterile artificial CSF (aCSF; Harvard Apparatus, AH 59-7316), 0.11 µl/h for 28 d (Alzet model #1004). In experiment 3, the mitotic inhibitor, AraC (Sigma; 33 µg/µl), was added to the BrdU/aCSF solution at the time of ICV cannula implantation; age-matched control animals received BrdU vehicle solution alone.

The use of BrdU as a marker of cell proliferation in the female rat AVPV was validated in a previous study, which found that 2 h after a systemic BrdU injection, virtually all BrdU-ir cells expressed proliferating nuclear cell antigen (Mohr et al., 2016). Pilot studies on ICV delivery of BrdU and AraC showed that: (1) in rats that received ICV BrdU for four weeks during puberty, abundant BrdU-labeled cells were observed in periventricular regions, but not in more lateral regions, including the medial amygdala; (2) in rats that received peripheral BrdU injections and ICV AraC, AraC did not affect the number of BrdU-labeled cells in the medial amygdala, but did decrease the number of BrdU-labeled cells in periventricular regions. These results indicate that, when administered ICV, BrdU is incorporated into proliferating cells only in periventricular cytogenic niches, and similarly that ICV AraC inhibits cell proliferation only in periventricular cytogenic niches. Furthermore, these results suggest that areas other than those surrounding the ventricles are not involved in the effects of AraC on the LH surge.

### Perfusions and tissue processing

Rats were killed with an overdose of sodium pentobarbital (90 mg/kg, i.p.) and transcardially perfused with 4% paraformaldehyde. Brains were removed, postfixed in 4% paraformaldehyde overnight, then switched to 30% sucrose in phosphate buffered saline until sectioning. Brains were coronally sectioned at 30 μm on a cryostat and processed per previous reports (Ahmed et al., 2008) and standard protocols. A one-in-four series of sections was mounted and Nissl-stained for reference.

### Single-label BrdU immunohistochemistry

Single-label immunohistochemistry to detect BrdU was performed on a one-in-four series of free-floating sections according to published protocols (Ahmed et al., 2008; Mohr and Sisk, 2013), with minor modifications, i.e., no formamide or nickel was used and a blocking step was added using AffiniPure Fab Fragment donkey anti-rat IgG (Jackson ImmunoResearch, catalogue number 712-007-003, 12 µg/ml; in TBS containing 0.1% Triton X-100 and 3% donkey serum; Jackson ImmunoResearch). The primary antibody was a rat monoclonal primary anti-BrdU (Serotec, MCA2060; 1 μg/ml), and the secondary was Biotin-SP-conjugated AffiniPure donkey anti-rat IgG (Jackson ImmunoResearch; 712–065-150; 2 μg/ml).

Using brightfield illumination on an Olympus BX51 microscope equipped with Neurolucida (MBF Bioscience), the outline of the AVPV was first traced from adjacent Nissl-stained sections. The traces were superimposed onto the BrdU-ir sections, and BrdU-ir cells were counted bilaterally in four anatomically matched sections of the AVPV.

BrdU-positive control tissue was used to confirm BrdU-immunoreactivity, and consisted of tissue from 1.5- to 2-month-old rats whose dam had received BrdU (200 mg/kg) during the latter end of gestation when the rate of embryonic neurogenesis is high. In addition, controls that excluded primary and secondary antibodies were run using experimental tissue that did not contain the AVPV (to conserve the small number of AVPV sections), and only minimal or no nonspecific background staining was observed. This control tissue was included in all other immunohistochemical procedures described below.

### Double-label BrdU and Fos immunofluorescence

BrdU and Fos were colocalized using immunofluorescence to detect activated cells. Previously published protocols were used (Mohr and Sisk, 2013) with the addition of a 10 min rinse in 0.1% sodium borohydride at the beginning of processing to reduce autofluorescence. The primary antibodies used were monoclonal rat anti-BrdU (Serotec, MCA2060; 1 µg/ml) and polyclonal rabbit anti-c-Fos (Santa Cruz, sc-52; 0.02 µg/ml). The secondary antibodies used were Biotin-SP-conjugated AffiniPure goat anti-rat (Jackson, 112-065-003; 1.3 µg/ml), Cy3-conjugated AffiniPure Goat anti-rabbit (Jackson, 111-165-144; 1.5 µg/ml). Cy2-conjugated streptavidin (Jackson, 016-220-084; 1.8 µg/ml) was used to visualize BrdU-immunoreactivity. Sections were mounted onto slides and coverslipped using SlowFade Gold antifade reagent (Life Technologies, catalogue number S36936).

Twelve sections spanning the AVPV and rostral and caudal regions were taken from a one-in-four series and processed, and then four sections of the AVPV, anatomically matched across animals, were analyzed for BrdU/Fos colocalization. An Olympus BX51 microscope equipped with epifluorescence illumination and an MBF Bioscience Image Stack Module for Neurolucida were used to first trace the outline of the AVPV from adjacent Nissl-stained sections, and then the traces were superimposed onto the fluorescent sections. Z-stacks were taken using the MBF Bioscience Image Stack Module for Neurolucida at 0.5-μm steps spanning the *z*-axis of BrdU-ir within the tissue using an UPlanSApo 40× (0.9 NA) objective and epifluorescence filters to visualize BrdU-positive (FITC, EX480, BS505, EM535) and Fos-positive (TRITC, EX535, BS565, EM610) cells. The macroview was used to insure both accurate localization and lack of overlap of the stacks. Colocalization was confirmed using the 3D Visualization module to rotate images. Neurolucida software was used to quantify cells using a modified fractionator method. Minimal contrast adjustments were made to the images, but images were otherwise unaltered.

### Double-label BrdU/ERα or BrdU/PR immunofluorescence

Sequential double-label immunofluorescence was used to colocalize BrdU and ERα or BrdU and PR. Tissue was rinsed in 0.05M TBS between all steps. Sections were incubated in 0.1% sodium borohydride for 10 min to reduce autofluorescence, then blocked in 1% hydrogen peroxide, 20% normal goat serum and 1% bovine serum albumin in TBS for 1 h. For ER localization, the primary antiserum was rabbit anti-ERα (Millipore, C1355; 0.04 µg/ml) for 48 h at 4°C. For PR localization, tissue was incubated in rabbit polyclonal antiserum directed against human PR (Dako, A009801-1; 1.2 µg/ml) for 72 h at 4°C. Following incubation in primary antisera, tissue was rinsed and transferred to biotinylated goat-anti-rabbit IgG (Vector, BA-1000; 1.5 µg/ml) for 1 h, then avidin/biotinylated enzyme complex reagent (ABC Elite kit; Vector Laboratories) for 1 h, then biotinyl tyramide (1:500 dilution with 1× amplification buffer from TSA Biotin System kit, PerkinElmer, NEL700A001KT), and finally in Cy3-conjugated streptavidin (Jackson, 016-160-084; 3 µg/ml) for 1 h. Tissue was then labeled for BrdU. After 30 min incubation in 2 N HCl at 37°C, tissue was rinsed in borate buffer (pH 8.5) for 10 min, and then incubated with BrdU monoclonal rat anti-BrdU (48 h at 4°C, then 1 h in Cy2-conjugated goat anti-rat IgG; Jackson, 112-225-167; 1.5 µg/ml).

Microscopic analyses of BrdU and ERα or BrdU and PR were performed as described above for colocalization of BrdU and Fos. Data are expressed as percentage of BrdU-ir cells that were also ERα- or PR-positive.

### Triple-label BrdU/NeuN/GFAP immunofluorescence

Triple label immunofluorescence for BrdU, the mature neuronal nucleus marker (NeuN), and the astrocyte marker, GFAP, was performed on a one-in-four series of sections per animal as described above. Primary antibody incubations were for 48 h at 4°C in a cocktail containing monoclonal rat anti-BrdU (Serotec, catalogue number MCA2060; 1 µg/ml), monoclonal mouse anti-NeuN (Millipore Bioscience Research Reagents, catalogue number MAB377, clone A60; 1 µg/ml), and polyclonal rabbit anti-GFAP (Dako, catalogue number Z0334; 0.58 µg/ml) in TBS containing 0.3% Triton X-100, 2% goat serum. Sections were incubated for 2 h in a secondary antibody cocktail solution containing biotin-SP-conjugated AffiPure goat anti-rat (Jackson, catalogue number 112 065 003; 1.3 µg/ml), Alexa Fluor 635 goat anti-mouse (Life Technologies, catalogue number A-31574; 4 µg/ml) and Cy3-conjugated AffiniPure Goat anti-rabbit (Jackson, catalogue number 111 165 144; 3 µg/ml), then for 1 h in Cy2-conjugated streptavidin (Jackson, catalogue number 016 220 084; 1.8 µg/ml), and then sections were mounted onto glass slides and coverslipped as above. Microscopic analysis to determine colocalization was performed as described above using a FITC filter to visualize BrdU, TRITC filter to visualize GFAP, and far red filter to visualize NeuN.

## Results

### Experiment 1. Pubertally born cells in the AVPV are activated by hormones

**Figure 1.**
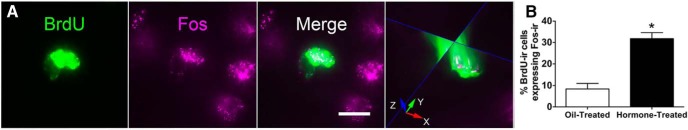
Pubertally born cells were activated by estradiol and P treatment. ***A***, Representative images of a pubertally born cell that was also Fos-ir after a hormone-induced LH surge in adulthood. Images are a maximum intensity projection of a Z-stack (0.5-μm step) captured using the MBF Bioscience Image Stack Module. Scale bar, 10 μm. An orthogonal view (right) through the middle of the cell confirms colocalization; red arrow, *x* plane; green arrow, *y* plane; and blue arrow, *z* plane. ***B***, Hormone induction of the LH surge caused a significant increase in the percentage of pubertally born BrdU-ir cells that express Fos-ir (*p ≤ 0.0001). The graph represents mean ± SEM; for oil treated, *n* = 6, for hormone treated, *n* = 7.

BrdU and Fos were identified in AVPV sections using double-label immunofluorescence (Fig. 1*A*). The average (±SEM) total number of BrdU-ir cells/rat examined was 218.8 ± 37.6. In the vehicle-treated animals only 8 ± 3% of BrdU-ir cells in the AVPV were Fos-positive, whereas in the estrogen/P-primed animals, 32 ± 3% of BrdU-ir cells in the AVPV were immunoreactive for Fos during the time of the expected LH surge (Fig. 1*B*; *F*_(1,11)_ = 35.676, *p* ≤ 0.0001). Hormone induction of the LH surge resulted, therefore, in an ∼4-fold increase in the proportion of pubertally born AVPV cells that were activated, indicating that a large subset (one third) of these cells are capable of responding to estradiol and P (Cohen’s *d* = 3.35).

### Experiment 2. Some pubertally born AVPV cells express ERα, but none express PR

**Figure 2.**
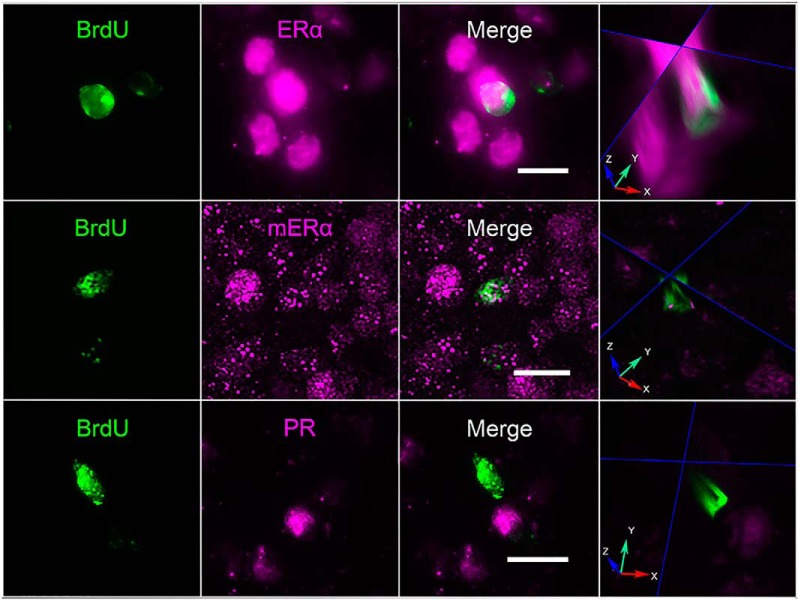
Some pubertally born cells were ERα-ir, but none were PR-ir. Z-stack images in the AVPV of BrdU-ir cells colocalized with nuclear (top panel) and membrane-associated ERα-ir (middle panel). BrdU did not colocalize with PR (bottom panel). BrdU, ERα, mERα, PR, and merge images are maximum intensity projections of 30 images (15 μm thick, 0.5**-**μm step). White scale bars, 10 μm. In the column on the right, orthogonal views through the middle of the cells confirm colocalization (top two panels) or lack of colocalization (bottom panel); red arrow, *x* plane; green arrow, *y* plane; and blue arrow, *z* plane. Fluorescent labeling for BrdU/ERα performed on tissue from four ovariectomized and vehicle-treated rats; labeling for BrdU/PR was performed on tissue from four ovariectomized and estradiol- and P-treated rats.

BrdU and ERα were identified in AVPV sections using double-label immunofluorescence. The average (±SEM) total number of BrdU-ir cells/rat examined was 166.6 ± 37.8. Approximately 9 ± 1% of pubertally born cells in the AVPV displayed nuclear ERα-immunoreactivity (Fig. 2). Additionally, another 6 ± 1% of BrdU-ir cells had ERα labeling that was not nuclear, but punctate and appeared to be membrane ERα (Fig. 2). No pubertally born BrdU-ir cells were immunoreactive for PR in the AVPV, although PR-positive, BrdU-negative cells were abundant (Fig. 2).

### Experiment 3. Addition of cells to the AVPV and SCN continues into adulthood. Inhibition of cell proliferation decreases cells added to AVPV and SCN during either puberty or adulthood

The day of vaginal opening, a peripheral indicator of the start of puberty in female rats, did not differ between animals treated with AraC or vehicle solution during puberty (control, 35.6 ± 0.9 d of age, AraC, 36.5 ± 1; *p* > 0.5). Animal weights changed as a result of age but not treatment (in grams, at the time of killing: pubertal control, 194.6 ± 6.3, pubertal AraC, 190.4 ± 10.0; adult control 221.6 ± 4.1, adult AraC, 212.4 ± 3.6; ANOVA significant effect of age, *p* < 0.001; no effect of treatment, *p* = 0.26; *n* = 7–8/group). Daily observations indicated that general health was unaffected by ICV AraC treatment.

**Figure 3.**
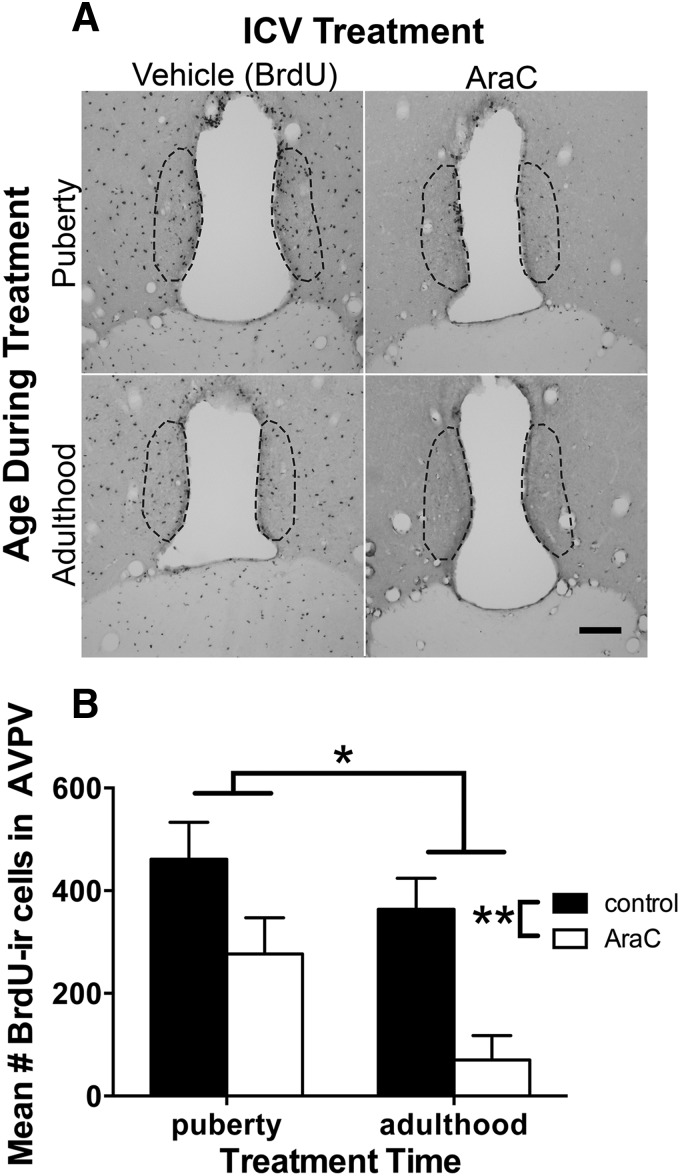
Fewer cells were added to the AVPV during adulthood than during puberty and the mitotic inhibitor, AraC, reduced the number of BrdU-ir cells in the AVPV. ***A***, Representative images show BrdU-ir cells in the AVPV of female rats treated with vehicle (BrdU in aCSF) or AraC (in BrdU and aCSF vehicle) during puberty or adulthood. Scale bar, 100 μm. ***B***, Graph depicts the quantitative results showing that on average, more cells were added to the AVPV during puberty than adulthood (main effect of age during treatment, *p = 0.017) and that on average, AraC reduced the number of BrdU-ir cells added to the AVPV (main effect of treatment, **p = 0.001). Graph represents mean ± SEM of the total number per animal of BrdU-ir cells in four anatomically matched sections through the AVPV; *n* = 7 for pubertal control, *n* = 5 for pubertal AraC, *n* = 6 for adult control, *n* = 7 for adult AraC.

The first key finding was that cells were added to the AVPV during puberty, as previously observed, but cells were also added to the AVPV during adulthood (Fig. 3*A*). Comparing the overall mean number of BrdU-ir cells, there were ∼41% more pubertally born cells than adult born cells (Fig. 3*B*; main effect of age during treatment, *F*_(1,21)_ = 6.687, *p* = 0.017; Cohen’s *d* = 0.88, large effect size), indicating that cell addition to the AVPV declines between puberty and adulthood. Next, as expected, inhibition of cell proliferation with ICV AraC treatment reduced the mean number of BrdU-ir cells in the AVPV, by an overall average of 58% (Fig. 3*B*; main effect of treatment, *F*_(1,21)_ = 16.648, *p* = 0.001; Cohen’s *d* = 2.1, large effect size). We did not have enough power to detect an interaction between AraC treatment and age (*F*_(1,21)_ = 0.8566, *p* = 0.3652), however visual inspection of the data suggests that AraC treatment was more effective at inhibiting cell proliferation during adulthood than during puberty (81% vs 40% knockdown of BrdU-ir cells compared to controls in adult- vs pubertal-treated rats, respectively).

Finally, quantification of BrdU-ir cells in the two rostral SCN sections available for analysis revealed a similar pattern of results as seen in the AVPV: (1) more cells are added to the SCN during puberty (mean ± SEM = 104.8 ± 21.4, pubertal-vehicle control) than in adulthood (68.6 ± 9.4, adult-vehicle control; main effect of treatment age, *F*_(1,16)_ = 8.945, *p* = 0.009; Cohen’s *d* = 0.98); and (2) AraC treatment reduced the number of BrdU-ir SCN cells (main effect of treatment, *F*_(1,16)_ = 6.732, *p* = 0.019; Cohen’s *d* = 1.71), whether administered during puberty (75.0 ± 21.9, pubertal-AraC) or in adulthood (14.6 ± 4.4, adult-AraC).

### Experiment 3. Adult-born AVPV cells can be activated. A subpopulation of both pubertally born and adult-born AVPV cells becomes neurons or astrocytes

**Figure 4.**
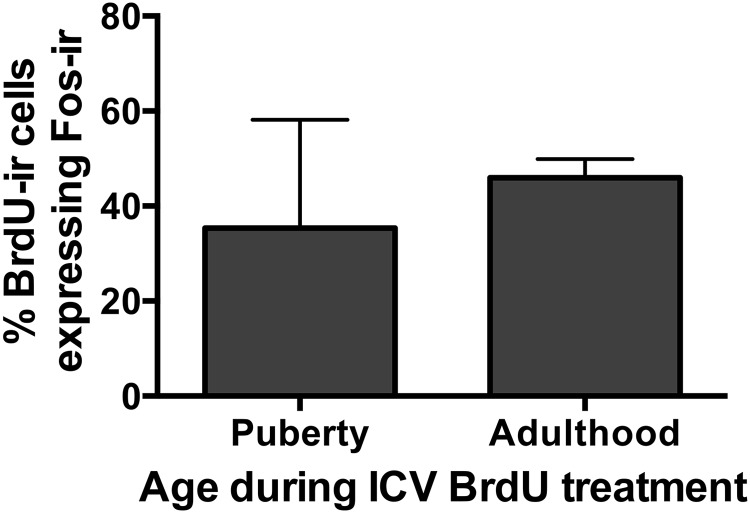
Both pubertally born and adult born cells in the AVPV were activated in adulthood by hormone treatments designed to induce an LH surge. The graph depicts the percentage of pubertally or adult-born (BrdU-ir) cells in the AVPV that also express Fos-ir during the time of the LH surge in adult female rats that were treated with BrdU during puberty or during adulthood. Data are presented as mean ± SEM; *n* = 3/age.

In the control groups (those that did not receive AraC), we asked whether AVPV cells born during adulthood are activated by hormone priming at the time of the expected LH surge, and whether the proportion of adult born cells that express Fos is similar to that of pubertally born cells. Approximately 40-50% of pubertally born and adult-born AVPV cells expressed Fos during the expected time of a hormone-induced LH surge, with no significant difference between groups (Fig. 4).

**Figure 5.**
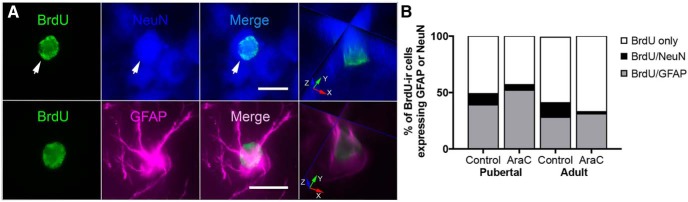
Some cells in the AVPV that were born during puberty or adulthood had differentiated by three weeks, with or without AraC-treatment. ***A***, Representative photomicrographs of pubertally born cells that were NeuN-ir or GFAP-ir. Images are maximum intensity projections of a Z-stack (0.5-μm step). Scale bars, 10 μm. Orthogonal views through the middle of the cells (right column) confirm colocalization; red arrow, *x* plane; green arrow, *y* plane; and blue arrow, *z* plane. ***B***, Histograms represent the mean proportion of BrdU-ir cells that express GFAP, NeuN, or only BrdU-immunoreactivity in animals receiving either vehicle (BrdU in aCSF) or AraC (AraC + BrdU in aCSF) ICV during puberty or adulthood (*n* = 2/age/treatment).

The proportion of pubertally or adult-born cells (BrdU-ir) that differentiate into neurons (BrdU-ir/NeuN-ir) or astrocytes (BrdU-ir/GFAP-ir), or were not identified as to either phenotype (BrdU-ir only) was determined by triple-label immunofluorescence (Fig. 5*A*). The average (±SEM) total number of BrdU-ir cells/rat examined was 105 ± 36 pubertal-control, 41 ± 23 pubertal-AraC, 164 ± 31 adult-control, and 21.5 ± 3.5 adult-AraC (*n* = 2/group). Across groups, the average percentage of BrdU-ir only cells was 55% (range 43-67%), BrdU-ir/GFAP-ir cells 37.5% (range 28-52%), and BrdU-ir/NeuN-ir cells 7.5% (range 2-13%; Fig. 5*B*). Thus, at the time point examined, just under half of late-born AVPV cells have differentiated into mature neurons or glia, with the majority of these differentiated cells of glial phenotype. Small sample sizes precluded statistical analysis. In addition, because BrdU was administered over a four-week period of time, BrdU-ir cell age at the time of tissue collection ranged from six to nine weeks, and we do not know whether or how cell age interacts with the potential for differentiation.

### Experiment 3. Inhibition of cell proliferation, during puberty or early adulthood, alters ovarian cyclicity and reduces and delays the LH surge

Inhibition of cell proliferation during either puberty or adulthood disrupted ovarian cyclicity as determined by monitoring of vaginal cytology for two weeks after discontinuation of AraC treatment. On average, pubertal and adult control rats, respectively, had 2.2 and 2 d with exclusively nucleated cells clearly indicative of proestrus, whereas pubertal and adult AraC-treated rats had on average only 0.6 and 1.2 proestrous days, respectively (main effect of treatment, *F*_(1,16)_ = 6.86, *p* = 0.019; Cohen’s *d* = 3.79).

**Figure 6.**
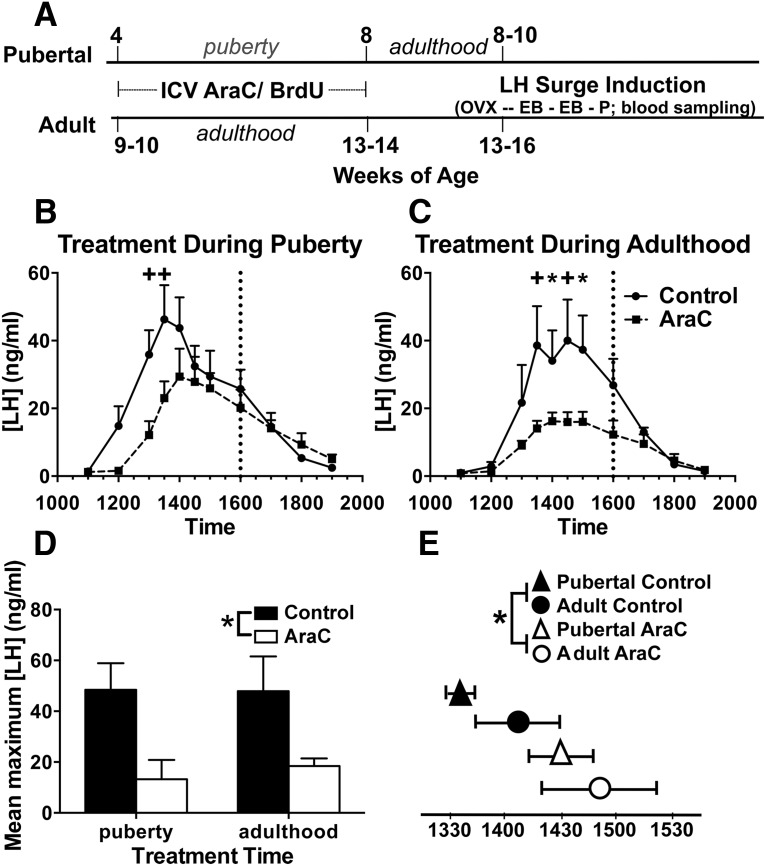
Knockdown of cell addition with AraC during puberty or adulthood reduced and delayed the LH surge. ***A***, Experimental timeline. Pubertal (4 week old) or adult (9-10 week old) female rats received ICV cannulae with osmotic minipumps containing AraC (33 μg/μl) and BrdU (4.5 μg/μl) in aCSF (AraC treatment group), or just BrdU in aCSF (control treatment group). Females were ovariectomized (OVX) after the end of ICV infusion and then primed with EB (10 μg in 0.05 ml sesame oil; s.c.) for two consecutive days at 10 A.M., and then P (500 μg in 0.1 ml sesame oil; s.c.) on the third day at 10 A.M. Blood sampling occurred on the same day as P injection, from 11 A.M. to 7 P.M. Pubertal (***B***) or adult (***C***) AraC treatment reduced the LH surge across time (*p* = 0.047). Dotted vertical lines indicate lights off. Symbols above points indicate significant differences observed with Fishers LSD *post hoc* comparisons. ***D***, AraC treatment reduced the maximum LH concentration (*p* = 0.022) compared with that observed in control animals. ***E***, The LH peak was delayed in AraC-treated animals (*p* = 0.05), irrespective of age during treatment. Graphs represent mean ± SEM; *n* = 4/group, except for pubertal-AraC (*n* = 3); *p ≤ 0.05, +*p* ≤ 0.01.

Inhibition of cell proliferation during either puberty or adulthood blunted and delayed the estradiol and P-elicited LH surge (Fig. 6). A two-way ANOVA (treatment × age during treatment) with repeated measures (LH concentrations at each time point) revealed that, compared with vehicle, AraC reduced the overall LH concentration at the time of the surge (Fig. 6*B*,*C*; *F*_(1,11)_ = 4.982, *p* = 0.047). Age during treatment (pubertal vs adult) did not affect overall LH concentrations (*F*_(1,11)_ = 1.228, *p* = 0.291), and there was no interaction between treatment and age during treatment (*F*_(1,11)_ = 0.162, *p* = 0.695). Area under curve calculations revealed that AraC treatment during puberty or during adulthood reduced the LH surge by 17% and 21%, respectively (data not shown). AraC treatment significantly reduced the average maximum LH concentration by 50% (Fig. 6*D*; two-way ANOVA, main effect of treatment, *F*_(1,11)_ = 7.080, *p* = 0.022; Cohen’s *d* = 1.4). Inhibition of cell proliferation delayed the timing of the LH peak by ∼50 min (two-way ANOVA, main effect of treatment, *F*_(1,11)_ = 4.82, *p* = 0.05; Cohen’s *d* = 1.1). The LH peak occurred at 1352 h ± 16 min in control-treated animals (collapsed across age during treatment), whereas in AraC-treated animals the LH peak occurred at 1439 h ± 15 min (Fig. 6*E*).

## Discussion

We provide evidence that postnatally born periventricular cells are functional components of hormone-responsive, reproductively relevant neural circuitry that controls adult GnRH/LH release, a process critical to ovulation. First, we discovered that ∼40% of AVPV cells born during puberty and adulthood are activated during a hormone-induced LH surge, suggesting that they may play a role in regulating the surge. Second, we demonstrated that inhibiting cell proliferation, during either puberty or adulthood, decreases the number of pubertally and adult-born AVPV and SCN cells, alters ovarian cyclicity, and blunts and delays the LH response to ovarian hormones, indicating that new cells are necessary for the full LH response. Our findings suggest a causal link between postnatal renewal of populations of hypothalamic cells and the integrity of neuroendocrine processes vital to female reproduction.

Our primary focus was on late-born cells in the AVPV, because estrogen action in the AVPV is both necessary and sufficient for hormone induction of the LH surge (Petersen and Barraclough, 1989; Radovick et al., 2012). However, we cannot rule out the possibility that pubertally or adult-born cells in other brain regions may also contribute to the generation of the GnRH/LH surge. AraC treatment could not be targeted specifically to the AVPV, because the location of the progenitor cells destined for the AVPV is unknown. The third ventricle and circumventricular organs are now known to be cytogenic niches for production of new brain cells in adulthood, and it is likely that at least some late-born AVPV cells migrate there from ventricular proliferative zones (Pérez-Martín et al., 2010; for reviews, see Bennett et al., 2009; Migaud et al., 2010; Cheng, 2013). Therefore, we chose ICV as the route of AraC administration for the current studies, which could reduce addition of cells to any region in which new cells originate in ventricular proliferative zones. Indeed, our preliminary examination of BrdU-ir cells in the SCN provides the first evidence that new cells are added during puberty and in adulthood to the SCN, and that the progenitor cells are in periventricular regions reached by ICV AraC. Thus, the current study provides evidence that late-born AVPV and SCN cells contribute to regulation of the magnitude and timing of the LH surge, a parsimonious conclusion consistent with the well-established functions of these two brain regions. However, other cell types or brain regions affected by AraC could be involved as well, e.g., tanycytes in the lining of the 3rd ventricle and the median eminence that regulate GnRH release (Prevot et al., 2007), or cells in the arcuate nucleus involved in negative feedback regulation of tonic GnRH/LH secretion (Blake et al., 1974; Blake, 1977; Kauffman et al., 2007). However, if AraC disrupted cell addition to the arcuate nucleus in the present study, it would seem unlikely to account for the observed blunting of the LH surge, as disruption of negative feedback would predict higher baseline (presurge) levels of LH in AraC-treated rats, which were not observed in this study. Regardless, if late-born cells in brain regions other than the AVPV and SCN are ultimately found to be involved in regulation of the LH surge, such evidence would corroborate the idea proposed here that ongoing renewal of hypothalamic cells is integral to female reproduction across the lifespan.

Mitotic inhibition decreased ovarian cyclicity and altered the LH surge, but did not eliminate either. Although proliferation was decreased substantially by AraC in the present studies, it was not completely eliminated, especially during puberty. Therefore, future experiments should determine the impact of completely blocking cell addition up to the time of induction of a surge. In addition, it will be important to determine whether interfering with cell proliferation has consequences on fertility, i.e., a decreased potential to become pregnant, given the disrupted cycles, or perhaps decreases in the number of ruptured follicles and therefore fewer offspring due to the lower level of LH secreted.

The lower number of proestrous days and the nearly 50% reduction in maximum LH release caused by AraC treatment are not likely the result of untoward side effects of AraC treatment. Kokoeva and others (Kokoeva et al., 2005), using higher doses of AraC than in the present study, demonstrated that ICV AraC is not cytotoxic, as has been reported using other paradigms (Sanz-Rodriguez et al., 1997), because following ICV AraC, no cell degeneration was detected in the brain parenchyma as determined by the cell death marker Fluoro-Jade. In the present study, body weight and overall health of the AraC-treated rats were normal, indicating that AraC was not merely degrading hypothalamic function, but was indeed acting as a mitotic inhibitor to reduce proliferation of neuronal precursor cells that give rise to neurons and glia that ultimately regulate the LH surge.

Approximately 10-13% of pubertally or adult-born AVPV cells were identified as neurons (BrdU/NeuN) and 30-40% as astrocytes (BrdU/GFAP). Pubertally born neurons could conceivably be kisspeptin neurons, given that the number of kisspeptin neurons in the AVPV markedly increases from P20 to adulthood, and that AVPV kisspeptin neuron signaling is required for estrogen positive feedback (Mayer et al., 2010). However, we were not able to determine whether this is the case because immunohistochemical labeling of kisspeptin protein in the rat AVPV requires pretreatment with colchicine (Franceschini et al., 2013). Newly born astrocytes might be among those that release neuroprogesterone in response to estrogen, an event critical for estrous cyclicity (Micevych et al., 2003). Because astrocytes in the prepubertal hypothalamus are not estrogen responsive (Kuo et al., 2010), and estrous cyclicity is established during puberty, we speculate that the higher number of astrocytes added during puberty than adulthood observed in this study may reflect the essential role of astrocytes in pubertal maturation of neuroendocrine positive feedback. Many of the late-born AVPV cells that were not identified as either neurons or astrocytes in this study are likely to be microglia, which could also play a role in regulation of cyclic hormone release (Mohr et al., 2016). Some late-born cells may remain undifferentiated, perhaps performing a unique role in hypothalamic function, or may require additional signals to promote differentiation (e.g., exposure to males, pregnancy). Pubertally and adult-born neurons and glia may constitute a unique, as yet unidentified, cohort of cells that subserve the crucial functions of this evolutionarily essential brain region.

Many pubertally born cells were activated by hormone priming with estradiol and P. Only a small subpopulation of pubertally born cells was steroid hormone receptor-positive, suggesting that the hormones acted indirectly through older, hormone receptor-positive cells, then activating the Fos response in the newly born cells. The AVPV is rich in ERα-expressing cells (DonCarlos et al., 1991; Smith, 2013), including both neurons and astrocytes (Langub and Watson, 1992), whereas only ∼10% of pubertally born cells in the AVPV displayed nuclear ERα-immunoreactivity in this study. Even with the addition of the 6% of pubertally born cells in the current study that had distinct membrane ERα-immunoreactivity, the proportion of pubertally born, ERα-expressing cells is lower than expected. Whether the pubertally born ERα-expressing cells are neurons or glia remains unknown. Although all PR-positive cells in the AVPV also express ERα (Blaustein and Turcotte, 1989; Warembourg et al., 1989) not all ERα-expressing cells are PR-positive; relatively few pubertally born cells were ERα-positive, which may explain why none of them were PR-ir.

The number of cells added to the AVPV was higher during puberty than in adulthood, and may reflect enhanced plasticity during this developmental phase (Juraska et al., 2013), when new social, behavioral, and neuroendocrine repertoires are acquired. While the mechanisms responsible for pubertal cell proliferation and survival are not fully known, earlier studies demonstrated that ovarian hormones drive the female-biased sex difference in the number of pubertally born AVPV cells, thus promoting the genesis and/or survival of pubertally born AVPV cells in females (Ahmed et al., 2008). A decline in cytogenesis from puberty to adulthood has been observed in other brain regions, including the dentate gyrus of the hippocampus (Okamoto et al., 2012), the nucleus accumbens, amygdala (Saul et al., 2014), hypothalamus (Haan et al., 2013; Zhang et al., 2017) and the prefrontal cortex (Staffend et al., 2014), and may reflect a general decline in cell renewal across the lifespan. If so, age-related waning in renewal of AVPV cells may contribute to a decrease in estrogen responsiveness and disruption of ovarian cyclicity in middle age. Indeed, in addition to depletion of follicles in the ovary, reproductive senescence has been attributed to altered hypothalamic signaling (Downs and Wise, 2009), as has aging in general (Zhang et al., 2017). We propose that ongoing postnatal addition of AVPV and SCN cells supports reproductive function during fertile periods of life, and that an age-related decrease in cell renewal is one mechanism underlying reproductive senescence.

Establishing the function and incorporation of pubertally or adult-born cells into existing neural circuits has been a major challenge facing investigations of the neuro- and gliogenesis that continue beyond early development (Cameron and Glover, 2015; Paredes et al., 2016). Although pubertal and adult neurogenesis has been recognized as an ongoing process that introduces new cells to the dentate gyrus and olfactory system, general acknowledgment of the functional relevance of addition of cells to other brain regions has lagged. This lack of recognition persists despite numerous reports demonstrating adult or pubertal cell addition in the hypothalamus (Kokoeva et al., 2005; Ahmed et al., 2008; Migaud et al., 2010; Pérez-Martín et al., 2010; Cheng, 2013; Mohr and Sisk, 2013; Chaker et al., 2016; Mohr et al., 2016), amygdala (Ahmed et al., 2008; Fowler et al., 2008; Saul et al., 2014; Jhaveri et al., 2017), cerebral cortex (Snyder et al., 2009), and basal ganglia (Ernst et al., 2014; Staffend et al., 2014; Inta et al., 2015), among others. Furthermore, converging lines of evidence point to adult-born cells as functional components of hypothalamic circuitry involved in energy homeostasis (Kokoeva et al., 2005; Lee et al., 2012; Bless et al., 2014; Bless et al., 2016; Djogo et al., 2016; Rizzoti and Lovell-Badge, 2017). We now extend this growing body of evidence to include a function for late-born cells in a different hypothalamic role: reproduction. Our results serve to dispel the often-repeated dogma that incorporation of new cells is functionally limited to two regions, and provide evidence suggesting that pubertal and adult born cells functionally incorporate into a hypothalamic network of fundamental importance to survival of the species.

## Synthesis

Reviewing Editor: Margaret McCarthy, Univ of Maryland School of Medicine Decisions are customarily a result of the Reviewing Editor and the peer reviewers coming together and discussing their recommendations until a consensus is reached. When revisions are invited, a fact-based synthesis statement explaining their decision and outlining what is needed to prepare a revision will be listed below.

This is an interesting follow-up study on the authors' previous observations that neurogenesis in the hypothalamus continues well into the postnatal period and adulthood. The authors extended this observation to suggest that postnatal neurogenesis is important for the timing and amplitude of the ovulatory LH surge in female rats. The observations are novel and the manuscript clearly written (except for a few minor issues below).

The manuscript reports on findings indicating that new neurons and glia are added to the anteroventral periventricular nucleus (AVPV) during puberty and adulthood. A subset of these neurons respond to hormone treatment that stimulates the luteinizing hormone (LH) surge. Global inhibition of neurogenesis through ICV administration of a mitotic inhibitor decreases new neuron and glia numbers in the AVPV and blunts and delays the estradiol/progesterone-stimulated LH surge.

However, two major concerns cannot be overlooked. First, the authors conclude that addition of new cells to the AVPV throughout life is required for normal LH surge generation but this conclusion is too strong and not entirely supported by the data. As the authors indicate, the mitotic inhibitor, AraC could not be directed in a manner to influence only cells in the AVPV, as the locus for progenitor cells destined for the AVPV is unknown. Likewise, targeting AraC to proliferative zones would inevitably affect cells destined for loci other than the AVPV. However, the current data do not preclude the possibility that cells outside the AVPV are either responsible for or contribute to the observed effects. The only conclusion that can be definitively drawn from the AraC studies is that inhibition of new cell production in the brain leads to aberrant LH surge generation

Second, there is no functional significance associated with the addition of new cells to the AVPV.

The first question of specificity to the AVPV can be dealt with in one of two ways:

1) Control experiments demonstrating AraC did not impact peripheral (pituitary) and other central tissues (e.g., POA and the median eminence) relevant to the surge. This could be as easy as quantifying BrdU in other brain regions of already treated animals, or may require new experiments. If other key brain regions were investigated and the only area showing new cell birth was the AVPV, the conclusions could be stated a bit more strongly.

2) Rephrase the primary conclusion of the paper that the effects are due specifically to the AVPV and instead note that the effects are due to reduction of newly proliferating cells.

The question of functional relevance GnRH and kisspeptin challenges in AraC-treated animals would be minimally required to rule out a pituitary and GnRH system defect, respectively. This issue could also be dealt with by toning down the primary conclusions and making sure that any statements about effects are limited to those that the data demonstrate.

Lastly and although not necessary, what would make this an exceptional study would be to test the fertility of AraC-treated adult females. The surge has been dampened but not obliterated. Thus, the question is whether the dampened surge has an actual physiological consequence.

Other Concerns:

1) Many parts of the Methods are redundant - in some cases, the impetus for the work and specific methodology are repeated across sections. For example, the first full paragraph on p. 12 repeats procedures mentioned on p. 8. Likewise, having statistics scattered throughout the methods also provides redundancy as several statistical methods could be listed for more than one analysis. This sections might be more streamlined by starting with the broad timeline and manipulations for each study then having specific sections for each method and concluding with a data analysis section.

2) On p. 8, it is unclear why only 3 animals/group were explored for Fos in the AVPV when 7 pubertal and 6 adult control brains were collected.

3) What is the rationale for collecting brains 1 week after the end of BrdU administration? Is this enough time for cells to differentiate and mature? In figure 5, a substantial number of BrdU cells do not express NeuN or GFAP suggesting that many cells have likely not reached maturity. The gold standard for assuring differentiation has taken place is 2 weeks.

4) On page 18, bottom, it is reported that 41% more cells are born during puberty than in adulthood. This is somewhat misleading, as control and AraC groups are combined in this 41%. If comparing pubertal and adult controls, much more similar numbers of cells are born at each time point.

5) P. 19, bottom of 1st para - while there is a main effect of AraC on the number of BrdU cell observed, the effect is driven by AraC being effective in adults and without effect in pubertal animals (i.e., figure 3B). This point should be further elaborated here and the findings considered in the context of AraC not significantly suppressing cell proliferation in pubertal animals.

6) Some of the double-labeling presented does not clearly show the double labeling indicated. In figure 2, for example, the BrdU cell appears to extend beyond the ER-alpha labling on the right side. Given that BrdU should be a nuclear label and ER-alpha might extend beyond the nucleus, it would be expected that a more precise overlap would be observed. Likewise, in figure 5, BrdU does not appear to co-label the GFAP cell, it looks more like a BrdU-labeled neuronal nucleus overlaying a glial cell.

7) In figure 6, the control animal for the pubertal group and the control animal for the adult group have LH surges that differ by an hour. Is it unusual for young adults to have earlier surges than adults a few weeks older?

MINOR:

1) P12, please specify if the perfused brains have been post-fixed.

2) P13, L6, Cy2-conjugated streptavidin is not a secondary antibody (L4)

3) P14, second to last line, “...in AraC- or vehicle-treated animals (n=2/age/treatment)”; these data are presented in Figure 5 and are anecdotal at best given the low sample size.

4) P16, the data in the first two lines should be presented in Results section.

5) Figure 1 should have representative images of oil vs. hormone-treated brain sections at a lower magnification to showcase the difference.

6) The statistical significance between control and AraC-treated pubertal animals in Figure 3B is a bit confusing. These two groups do not appear different due to large error bars and have no symbol to indicate significance, but are indicated as different in the legend.

7) Figure 7 is not very useful. The correlation likely describes the potency of mitotic inhibition (not specific to AVPV) and LH. The correlation is relatively weak and the message somewhat misleading, as this correlation can likely be achieved with a different brain region affected by AraC.
